# Can Wideband Absorbance Be Used in the Detection of Ossicular Chain Defects?

**DOI:** 10.22038/IJORL.2022.63837.3186

**Published:** 2022-09

**Authors:** Fulya Savran Turanoglu, Ozan Ozdemir, Cigdem Kalaycik Ertugay, Ozgur Yigit

**Affiliations:** 1 *Department of Otorhinolaryngology-Head and Neck Surgery, Arnavutkoy State Hospital, Istanbul, Turkey.*; 2 *Department of Otorhinolaryngology-Head and Neck Surgery, University of Health Sciences Istanbul Training and Research Hospital, Istanbul, Turkey.*

**Keywords:** Acoustic impedance tests, Middle ear, Ear ossicles, Hearing loss, Otitis media

## Abstract

**Introduction:**

We aimed to compare the effectiveness of wideband absorbance in detecting ossicular chain discontinuity with intraoperative findings.

**Materials and Methods:**

In this study, 58 ears from 38 patients with chronic otitis media (COM) were included. Twenty-six ears with perforation and intact ossicular chain were determined as Group 1, 12 ears with perforation and ossicular chain defects were determined as Group 2, and 20 ears with normal hearing and intact tympanic membrane were determined as Group 3. The comparison of the groups was made considering the static (non-pressure) absorbance analysis performed using wideband tympanometry.

**Results:**

When perforation sites were evaluated in Group 1 and Group 2; there were 12 anterior perforations, 7 posterior perforations, and 19 subtotal perforations. Air conduction thresholds in Group 2 were significantly (P<0.05) higher than in Group 1, as expected in pure tone audiometry. When wideband absorbance (WBA) measurements were evaluated in all 3 groups, no significant difference (P>0.05) was found between the frequencies 226 to 1000 Hz. WBA measurements at 8 frequencies between 1888-2311 Hz in Group 1 were significantly lower than Group 3 (P<0.05). WBA measurements at 4 frequencies between 3462-3886 Hz frequencies in Group 2 were significantly lower than Group 1 (P<0.05).

**Conclusions:**

Our findings concluded that a significant decrease in absorbance values in the narrow frequency range may be valuable in predicting ossicular chain defects.

## Introduction

Chronic otitis media (COM) affects 65-330 million people worldwide, especially in developing countries (1). Ossicular chain erosion affects approximately one third of COM cases and causes hearing loss (2). 

Since the ossicular chain is best determined during surgery, the risk of decrease in the success rate due to a possible ossicular chain defect and the possibility of reoperation should be explained to the patient detailly before the operation (3). 

For this reason, it is important to determine if there is an ossicular chain discontinuity in the preoperative period. Computed tomography (CT) is the most commonly used method for ossicular chain evaluation in the preoperative period. Sensitivity and specificity is 73.1% and 84.8% for CT respectively. (4). 

In addition to its low sensitivity and specificity, another disadvantage of CT  is the inevitable radiation exposure. Therefore, there is a need for another non-invasive diagnostic method that can guide the surgeon about the ossicular chain status. 

The determination of the air-bone gap with pure tone audiometry has great importance in predicting the pathologies of the ossicular chain. The presence of a distinct air-bone gap suggests ossicular chain pathology especially at 1000-2000 Hz (5).

Standard tympanometry is first introduced by Terkildsen et al. which is generally unsuccessful in detecting ossicular chain discontinuities. 

For example, a 226 Hz frequency probe tone cannot distinguish between normal middle ear and otosclerosis (6,7). Standard tympanometry is also insufficient in cases with tympanic membrane perforation.

Perforation, sclerotic plaques, and similar pathologies affecting the tympanic membrane, will be strongly reflected in the measured admittance even at a level that does not affect hearing thresholds. (8). The inadequacy of standard tympanometry has led researchers to determine a new method. (9).

In the wideband tympanometry (WBT) system, hundreds of tympanograms are brought together and displayed in 3D with a single and rapid pressure flow, in the range of 226-8000 Hz. (10). Unlike standard tympanometry with static absorbance measurement, non-pressurized measurements can be made in patients with a ventilation tube or perforated tympanic membrane, and acoustic energy absorbed by the middle ear can be measured (11). Otosclerosis, flaccid eardrum, ossicular chain disorders, and semicircular canal dehiscence can be determined more clearly with the absorbance graph, especially negative middle ear pressure and middle ear effusion in infants (11-14). 

In this study, we aimed to investigate the effectiveness of static absorbance data obtained by preoperative WBT in detecting ossicular chain discontinuity in patients scheduled for surgery due to chronic otitis media.

## Materials and Methods

***Ethical Approval***

The local Institutional Ethics Committee approved the study (No.609/27.02.2015). Relevant ethical guidelines were followed in all procedures performed, and all patients signed an informed consent form.

***Patients and study design***

This clinical study conducted between May 2015 and May 2017 in the department of otorhinolaryngology of our hospital. 58 ears from 38 patients who were planned to be operated due to COM were included. 

The diagnosis of COM was made with the presence of perforation and intermittent discharge for at least 3 months. Otoscopic and microscopic external ear canal and tympanic membrane examinations were performed in all patient and control groups. The external ear canal abnormality if existed, the size and location of the tympanic membrane perforation, the condition of the middle ear mucosa or the ossicular chain if visible, and any signs of infection were recorded.

All participants had temporal bone CT examination. CT evaluation was performed by a radiologist in the preoperative period. Pure tone audiometry was performed by using AC-40 Clinical Audiometry (Interacoustic, Middlfart, Denmark) and TDH-39 (Telephonics, USA) headphones in a soundproof room. Air and bone conduction thresholds were measured, and the air-bone gap was calculated for each patient. Static (non-pressure) absorbance analysis was performed with WBT for both ears (perforated and intact) of all patients.

During the operation, the condition of the middle ear mucosa, malleus, incus, stapes and ossicular conduction, presence of cholesteatoma, granulation, polyps, and sclerotic plaques were recorded detailly. Patients under 12 and over 65 years of age, have granulation and cholesteatoma tissue in the middle ear on microscopic examination or soft tissue density in the middle ear or mastoid cavity on CT or sclerosis around the ossicular chain, patients who had hearing loss over 60 dB in pure tone audiometry examination, had the previous surgery, had an anomaly of the external auditory canal, had attic perforation and attic region bone defect were excluded. Erosion of the long arm of the incus and/or lenticular process was observed in 9 of 12 patients with ossicular chain defects.

The stapes head and crus erosion was detected in only one patient, while both the long arm of the incus and the head of the stapes were eroded in 2 of the remaining patients.

The ears were divided into three groups. 26 ears with central perforation of the tympanic membrane, intact and mobile ossicular chain during the operation and have normal middle ear mucosa were determined as Group 1 (perforated group). 

12 ears with perforation in the tympanic membrane, with an ossicular chain defect that blocks ossicular movement transmission during the operation (incus long arm erosion, stapes head, lenticular process erosion, incus and stapes erosion, etc.), without any additional pathology in the middle ear mucosa and around the ossicular chain were determined as Group 2 (perforated + ossicular chain defect group). 

20 ears with no perforation, no mastoid and middle ear pathology on CT examinations, no intraoperative ossicular chain defect, <10 dB air-bone gap in pure tone audiometry, and have normal thresholds (air conduction better than 25 dB HL) were determined as Group 3 (intact tympanic membrane group).

*Wideband absorbance measurement*

WBT measurements were conducted by using Titan® tympanometer (Interacoustics, Denmark). The WBT device tests the middle ear with the frequency range of 226 Hz to 8000 Hz with a single measurement. The probe tone used in the measurement is designed as a narrow band click with a stable output in the frequency spectrum between 226 Hz and 8000 Hz. It is applied by sending a 21-click stimulus per second at an intensity of 100 dB peSPL in adults and 96 dB peSPL in newborns between 0-6 months. If we refer to the frequency plane as the “X” axis, the “Y” axis is expressed as absorbance. The energy absorbance results range from 1 (all sound energy absorbed by the middle ear) to 0 (all sound energy reflected by the middle ear).

*Statistical analysis*

Statistical analysis was performed using the IBM® SPSS 22.0 software (SPSS Corp.; Armonk, NY, USA). 

Kolmogorov-Smirnov test was used to determine whether the variables were normally distributed. Descriptive analysis was presented using median and mean±standard deviation for variables with a normal distribution. ANOVA (Tukey test) and the Mann–Whitney U test were used for unpaired group comparisons and the Kruskal–Wallis test was used for comparisons between more than two groups. Spearman Correlation Test was used to compare the measurement data with each other. A *p* value less than 0.05 was considered as statistically significant.

## Results

The mean age of the patients was 33 (ranging from 13 to 62). 58 ears were included in the study; 30 of them were right side and 28 were left side. When perforation sites were evaluated in Group 1 and Group 2; there were 12 anterior perforations, 7 posterior perforations, and 19 subtotal perforations (Table. 1).

Age, gender, and side distribution did not differ significantly in Group 1 (perforated group), Group 2 (perforated+ossicles chain defect group), and Group 3 (intact tympanic membrane group) (P>0.05). 

The sites and sizes of the perforations did not differ significantly (P>0.05) in Group 1 and Group 2 (Table. 1). 

For Group 1 and Group 2, the mean air-bone gap values in pure tone were significantly higher than Group 3 (P< 0.05). The air-bone gap value for group 2 was significantly (P<0.05) higher than Group 1 (Table. 1). 

**Table 1:** Demographic data of the patients

		**Group1**			**Group2**			**Group3**			p
		mean±sd / n-%		median	mean±sd / n-%		median	mean±sd / n-%		median	
Age		33.3±13.2		34	35.6±15.8		33.0	34.2±11.8		32.5	0.887k
Gender	male	12	46.2%		7	58.3%		11	55%		0.743x^2^
	female	14	53.8%		5	41.7%		9	45%		
Ear side	right	9	34.6%		8	66.7%		13	65&		0.063x^2^
	left	17	65.4%		4	33.3%		7	35%		
Perforation type	anterior	9	34.6%		3	25%					0.272x^2^
	posterior	3	11.5%		4	33.3%					
	anterior + posterior	14	53.8%		5	41.7%					
Perforation size		5.7±1.6		7.0	5.8±1.1		6.0				0.714m
Air conduction		40.5±11.6		39.0	52.9±14.1		55.0	14.5±6.2		12.5	0.000k
Bone conduction		14.1±8.3		13.0	18.9±10.7		17.5	5.3±5.3		5.0	0.000k
Air-bone gap		26.4±7.1		26.0	34±12.2		36.0	9.3±5.1		10.0	0.000k
							

There was no significant difference between the groups (P>0.05) in the measurements obtained at 35 frequencies between 226-1000 Hz in wideband absorbance (WBA) measurements (Table. 2). 

**Table 2:** Wideband absorbance results according to groups between 200-1000 Hz frequencies

**Hz**	**Group1**		**Group2**		**Group3**		**p**
	**mean** **±** **sd / n-%**	**median**	**mean** **±** **sd / n-%**	**median**	**mean** **±** **sd / n-%**	**median**	
226	0.18±0.13	0.13	0.32±0.33	0.14	0.28±0.24	0.22	0.290^k^
281	0.27±0.12	0.25	0.37±0.30	0.24	0.37±0.24	0.33	0.320^k^
324	0.23±0.12	0.22	0.33±0.29	0.21	0.34±0.24	0.28	0.266^k^
364	0.31±0.13	0.31	0.37±0.27	0.25	0.41±0.23	0.36	0.408^k^
408	0.35±0.14	0.35	0.39±0.26	0.29	0.45±0.23	0.41	0.316^k^
459	0.38±0.15	0.38	0.40±0.25	0.34	0.48±0.24	0.43	0.392^k^
500	0.40±0.17	0.39	0.40±0.25	0.35	0.51±0.24	0.45	0.300^k^
545	0.47±0.18	0.43	0.45±0.25	0.42	0.57±0.25	0.52	0.314^k^
578	0.42±0.20	0.38	0.40±0.27	0.34	0.54±0.28	0.48	0.210^k^
630	0.46±0.20	0.41	0.44±0.28	0.41	0.60±0.28	0.54	0.123^k^
667	0.50±0.21	0.45	0.49±0.28	0.48	0.63±0.26	0.61	0.177^k^
707	0.54±0.21	0.48	0.52±0.28	0.55	0.66±0.24	0.68	0.180^k^
749	0.57±0.22	0.53	0.54±0.27	0.59	0.68±0.22	0.69	0.224^k^
794	0.58±0.23	0.52	0.52±0.27	0.58	0.67±0.22	0.69	0.157^k^
841	0.57±0.24	0.51	0.51±0.28	0.57	0.67±0.22	0.77	0.145^k^
891	0.57±0.24	0.50	0.52±0.27	0.60	0.69±0.22	0.77	0.116^k^
944	0.58±0.25	0.52	0.52±0.29	0.57	0.69±0.22	0.80	0.154^k^
1000	0.58±0.24	0.54	0.56±0.27	0.62	0.73±0.22	0.84	0.081^k^

k: Kruskal-wallis test.

Similarly, no significant (P>0.05) difference was found at 30 frequencies between 1029-2997 Hz (Table. 3). In Group 1, the WBA measurements for 8 frequencies between 1888-2311 Hz were significantly lower than Group 3 

(P<0.05). In the WBA measurements performed at 8 frequencies between 1888-2311 Hz, the analysis showed no significant (P>0.05) difference between Group 2 and Group 3 (Table. 3).

**Table 3:** Wideband absorbance results according to groups between 1000-3000 Hz frequencies

**Hz**	**Group1**		**Group2**		**Group3**		**p**
	**mean** **±** **sd / n-%**	**median**	**mean** **±** **sd / n-%**	**median**	**mean** **±** **sd / n-%**	**median**	
1029	0.59±0.23	0.56	0.58±0.26	0.64	0.74±0.21	0.81	0.070^k^
1155	0.58±0.23	0.61	0.60±0.30	0.66	0.71±0.20	0.75	0.213^k^
1260	0.57±0.26	0.60	0.57±0.35	0.71	0.69±0.20	0.74	0.369^k^
1374	0.57±0.27	0.57	0.60±0.35	0.78	0.70±0.20	0.80	0.332^k^
1456	0.55±0.27	0.50	0.65±0.32	0.74	0.72±0.21	0.81	0.140^k^
1587	0.53±0.28	0.50	0.66±0.35	0.80	0.71±0.22	0.76	0.091^k^
1682	0.53±0.28	0.50	0.64±0.34	0.77	0.71±0.22	0.75	0.124^k^
1782	0.53±0.27	0.51	0.64±0.30	0.78	0.72±0.22	0.76	0.101^k^
1888	0.52±0.26	0.47	0.65±0.26	0.69	0.74±0.21	0.78	**0.034** ^k^
2000	0.52±0.27	0.45	0.61±0.28	0.60	0.72±0.21	0.76	**0.041** ^k^
2119	0.51±0.20	0.42	0.60±0.29	0.63	0.72±0.21	0.77	**0.043** ^k^
2245	0.54±0.26	0.50	0.62±0.28	0.66	0.74±0.20	0.79	**0.020** ^k^
2311	0.55±0.25	0.52	0.61±0.28	0.66	0.74±0.21	0.78	**0.035** ^k^
2448	0.60±0.26	0.61	0.54±0.28	0.61	0.73±0.22	0.79	0.115^k^
2520	0.61±0.25	0.62	0.56±0.28	0.59	0.73±0.23	0.79	0.148^k^
2670	0.60±0.24	0.64	0.53±0.27	0.59	0.69±0.23	0.77	0.173^k^
2828	0.59±0.24	0.63	0.51±0.26	0.55	0.65±0.22	0.64	0.350^k^
2997	0.58±0.22	0.57	0.48±0.23	0.52	0.62±0.22	0.64	0.263^k^

k: Kruskal-wallis test.

The WBA measurements performed at 4 frequencies between 3462-3886 Hz in Group 2, the frequencies were significantly lower than Group 1 (P<0.05). 

In Group 1, the WBA measurements performed at 4 frequencies between 3462-3886 Hz, the frequencies did not differ significantly (p>0.05) between Group 1 and Group 3 (Table. 4).

**Table 4:** Wideband absorbance results according to groups between 3000-8000 Hz frequencies

Hz	**Group1**		**Group2**		**Group3**		p
	**mean** **±** **sd / n-%**	**median**	**mean** **±** **sd / n-%**	**median**	**mean** **±** **sd / n-%**	**median**	
3084	0.58±0.22	0.56	0.48±0.22	0.50	0.61±0.22	0.63	0.272^k^
3268	0.59±0.22	0.58	0.47±0.23	0.50	0.60±0.22	0.61	0.307^k^
3462	0.59±0.24	0.62	0.39±0.23	0.33	0.55±0.22	0.57	**0.034** ^k^
3668	0.58±0.24	0.59	0.33±0.24	0.25	0.53±0.23	0.54	**0.010** ^k^
3886	0.48±0.31	0.44	0.16±0.36	0.16	0.43±0.31	0.42	**0.049** ^k^
4000	0.44±0.33	0.47	0.19±0.35	0.18	0.43±0.33	0.42	0.083^k^
4238	0.40±0.29	0.42	0.31±0.27	0.28	0.46±0.31	0.47	0.300^k^
4490	0.43±0.25	0.39	0.42±0.25	0.40	0.48±0.29	0.52	0.733^k^
4757	0.44±0.30	0.40	0.36±0.30	0.30	0.40±0.33	0.46	0.659^k^
5040	0.52±0.26	0.47	0.37±0.38	0.27	0.44±0.32	0.41	0.335^k^
5339	0.50±0.26	0.47	0.42±0.44	0.46	0.35±0.30	0.37	0.269^k^
5657	0.47±0.28	0.51	0.46±0.41	0.49	0.33±0.29	0.33	0.222^k^
5993	0.42±0.26	0.51	0.46±0.32	0.42	0.30±0.30	0.32	0.231^k^
6350	0.39±0.27	0.43	0.45±0.33	0.47	0.26±0.34	0.25	0.239^k^
6727	0.43±0.27	0.49	0.45±0.34	0.55	0.28±0.31	0.27	0.159^k^
7127	0.48±0.28	0.47	0.44±0.31	0.50	0.31±0.26	0.30	0.133^k^
7551	0.42±0.24	0.43	0.36±0.27	0.34	0.29±0.24	0.27	0.203^k^
8000	0.37±0.28	0.37	0.33±0.22	0.30	0.28±0.23	0.22	0.447^k^

There was a significant (p<0.05) positive correlation between the perforation size and the WBA measurements performed at 9 consecutive frequencies between 841-1059 Hz, 10 consecutive frequencies between 2311-2997 Hz, and also 5657 Hz and 5823 Hz.

When CT examinations and perioperative ossicular chain status were compared in group 2, the presence of defect was detected in only 3 (25%) of 12 patients with CT findings.

**Fig 1:**
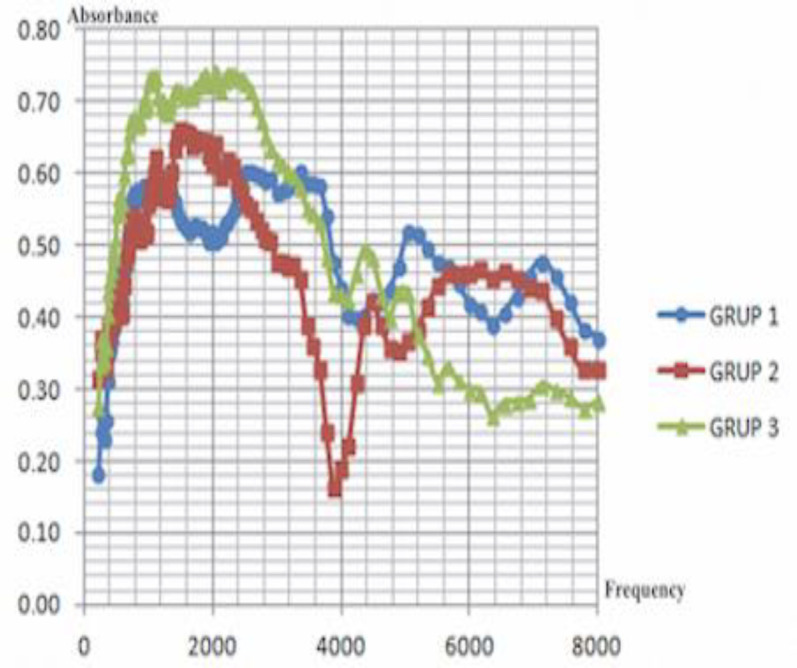
Average absorbance measurements by frequencies. Group 1 (Perforated group), Group 2 (Perforated + ossicular chain defect group), Group 3 (Intact membrane-Control group). Absorbance; % 0-1, Frequency; between 226-8000 Hz

The curve of mean absorbance values according to the frequencies in each of the 3 groups is shown in figure 1.

## Discussion

Wideband reflectance (WBR) is the ratio of the reflected power to the total power. It includes wideband acoustic immittance, wideband reflectance (WBR), and WBA measurements (15). WBA is a branch of tympanometry that quickly repeats a click stimulus to test the frequency range of 226 to 8000 Hz. Early studies focused on the analysis of reflectance data obtained from single frequency and multifrequency admittance tympanograms (16). Absorbance shows similar characteristics to traditional tympanometry with its morphological peak (17). Due to technological limitations in reflectance measurement and unclear data on its analysis, many studies focus on WBA (18). The clinicians are trying to prove that WBT can be used as a tool to examine middle ear function and diagnose middle ear diseases. However, there are some necessities such as knowing the effects of procedure depending variables, setting of clinical standards and determining validity and reliability. Based on these informations, we preferred to use WBA data in our study.

While the long-term success of surgery reaches 90% in cases with intact ossicular chains, it has been shown that this rate decreases in the presence of ossicular chain pathology (19). In patients planned for tympanoplasty, audiological examination and CT examinations are important to see the effects of the disease on the middle and inner ear. O'Reilly et al. were able to show that the ossicular chain was intact in 50% of their cases on CT (20). On the other hand, Thukral et al. found the sensitivity as 80.65% and the specificity as 46.67% in the detection of ossicular chain erosion in high-resolution CT (21). This low rate of specificity was similarly demonstrated by Keskin et al (22). In the evaluation of the ossicular system, malleus and incus pathologies can be easily distinguished on CT, while it is difficult to distinguish defects in the manubrium, the lenticular process of incus, and stapes (20). In our study, all patients had CT scan and its success rate for detecting ossicular chain discontinuity was found to be 25%. The studies mentioned above have shown that; additional objective tests that performed in addition to CT evaluation would increase the confidence of the surgeon.

With WBT, absorbance can be evaluated at peak pressure and ambient pressure (12). Since we studied on the perforated tympanic membrane, we were able to evaluate the absorbance data at ambient pressure in our study. In the presence of ossicular discontinuity, energy is absorbed in the middle ear but not transferred to the cochlea which causes a decrease in middle ear stiffness. The effect of reduction in middle ear stiffness was investigated in a cadaver study, and removing a 2 mm portion of the long arm of the incus revealed a significant notch in the reflectance pattern between 561-841 Hz. The notch disappeared upon surgical repair of the injury (23). Voss et al. measured ER patterns in four temporal bone cadavers. Incudostapedial joint disarticulation was surgically simulated in the cadavers and they reported a large reduction in ER below 1200 Hz as a result. (24). In the presence of tympanic membrane perforation, significantly lower energy reflectance values ​​were obtained below 841 Hz compared to higher frequencies (13). In another study conducted in cadavers, it was observed that the disarticulation created at the incudostapedial joint caused a sharp decrease in reflectance at a frequency below 1000 Hz. (25). However, all these cadaveric studies are related to chain defects with intact tympanic membranes. There is no study in the literature examining chain defects with tympanic membrane perforation.

In a cadaver study conducted by Voss et al., it was shown that the reflectance decreased at all frequencies with perforation, the most significant decrease was observed at frequencies below 2000 Hz, and this effect was mostly seen in perforations formed in small sizes (0.5 mm). As the perforation size increased, the reflectance at low frequencies systematically approached the normative value for reflectance (25). In our study, we found that the increase in perforation size at certain frequencies significantly increased the absorbance. 

In clinical studies evaluating different middle ear pathologies (otosclerosis, otitis media with effusion, tympanic membrane perforation, ossicular chain defect) together, the reliability of the data is low due to limited number of cases. Therefore, our study was superior to other studies in terms of design, as it was conducted in homogeneous groups that included more patients with a specific middle ear pathology (tympanic membrane perforation and ossicular chain defect). In our study, using WBA data in patients with COM with ossicular discontinuity, we found significantly low WBA values ​​at 4 frequencies between 3462-3886 Hz frequencies created a notch, and this was not detected in the group with intact ossicular chain. In the group with perforation and intact ossicular chain, WBA measurements at 8 frequencies between 1888-2311 Hz were significantly lower than the control group with intact membrane.

Although we had expected this effect of tympanic membrane perforation at lower frequencies, we found that the effect of perforation created a notch by reducing the absorbance at these frequencies. While planning our study, we excluded patients with intraoperative cholesteatoma, granulation tissue, and sclerosis as they would have an additional effect on the ossicular system. Thus, we were able to obtain a pure group with only perforation and ossicular chain defect, but our number of our patients decreased as ossicular chain problems were usually seen in chronic otitis media with cholesteatoma. In this regard, more valuable data can be obtained by increasing the number of patients or by conducting multicenter studies.

It was shown that the WBT absorbance value was higher in young adults (26). While the absorbance was found to be higher in men below 1000 Hz, it was found higher in women at high frequencies (27). It was also reported that absorbance values that vary according to gender might be related to the change in body mass (28). 

Although the gender and age distribution between the groups was homogeneous in our study, one of the limitations of our study was that we were not able to assess the data by gender, age, and body mass. In this study, a significant decrease in absorbance values ​​in a certain frequency range in COM cases with ossicular chain defects shows that WBT is a promising way to evaluate preoperative integrity of the chain. 

The fact that WBA values ​​can vary according to factors such as age, gender, body mass index, and middle ear volume suggests that measurements to be made by creating different middle ear pathologies in the same patient may provide more valuable information. For this reason, we believe that cadaver studies, in which different middle ear pathologies are created and measured in the same patient sequentially, can make a valuable contribution to the literature. However, although clinical studies involving more patients are necessary, middle ear tests with high sensitivity and specificity can be developed with a large amount of data available with WBT measurements. We believe that our study can contribute to the literature on this issue.

## Conclusions

Our findings showed a significant decrease in absorbance values corresponding to mid frequencies in a narrow frequency range that could be valuable in predicting ossicular chain defects. Further studies with a large number of cases are required to investigate these promising preliminary findings.

## References

[1] Monasta L, Ronfani L, Marchetti F, Montico M, Vecchi Brumatti L, Bavcar A (2012). Burden of disease caused by otitis media: a systematic review and global estimates. PLoS One..

[2] Sarmento KMA Jr, de Olivieira CACP, Sampaio ALL, Sales AF (2018). Erosion of the long process of the incus with incomplete ossicular discontinutiy in simple chronic otitis media: Should we reconstruct or leave it be?. Clin Otolaryngol..

[3] Sheikh R, Haidar H, Abdulkarim H, Aslam W, Larem A, Alsaadi A (2016). Preoperative Predictors in Chronic Suppurative Otitis Media for Ossicular Chain Discontinuity: A Cross-Sectional Study. Audiol Neurotol..

[4] Gül A, Akdağ M, Kiniş V, Yilmaz B, Şengül E, Teke M (2014). Radiologic and surgical findings in chronic suppurative otitis media. J Craniofac Surg..

[5] Jeng FC, Tsai MH, Brown CJ (2003). Relationship of preoperative findings and ossicular discontinuity in chronic otitis media. Otol Neurotol..

[6] Vaidya S, Sharma JK, Singh G (2014). Study of the outcome of tympanoplasties in relation to size and site of tympanic membrane perforation. Indian J Otolaryngol Head Neck Surg..

[7] Terkildsen K, Thomsen KA (1959). The influence of pressure variations on the impedance of the human ear drum. J Laryngol and Otol.

[8] Shanks JE (1984). Tympanometry. Ear Hear..

[9] Margolis RH, Van Camp KJ, Wilso RH, Creten WL (1985). Multifrequency tympanometry in normal ears. J Audiology..

[10] Kei J, Sanford CA, Prieve BA, Hunter LL (2013). Wideband acoustic immittance Measures: developmental characteristics (0 to 12 months). Ear Hear..

[11] Shahnaz N, Polka L (1997). Standard and multifrequency tympanometry in normal and otosclerotic ears. Ear Hear..

[12] Burdiek LM, Sun XM (2014). Effects of consecutive wideband tympanometry trials on energy absorbance measures of the middle ear. J Speech Lang Hear Res..

[13] Feeney MP, Grant IL, Marryott LP (2003). Wideband energy reflectance measurements in adults with middle-ear disorders. J. Speech Lang Hear Res..

[14] Sanford CA, Feeney MP (2008). Effects of maturation on tympanometric wideband acoustic transfer functions in human infants. J Acoust Soc Am..

[15] Voss SE, Allen JB (1994). Measurement of acoustic impedance and reflectance in the human ear canal. J Acoust Soc Am..

[16] Keefe DH, Levi E (1996). Maturation of the middle and external ears: acoustic power-based responses and reflectance tympanometry. Ear Hear..

[17] Liu YW, Sanford CA, Ellison JC, Fitzpatrick DF, Gorga MP, Keefe DH (2008). Wideband absorbance tympanometry using pressure sweeps: System development and results on adults with normal hearing. J Acoust Soc Am..

[18] Sanford CA, Hunter LL, Feeney MP, Nakajima HH (2013). Wideband acoustic immittance: tympanometric measures. Ear Hear..

[19] Santosh UP, Prashanth KB, Rao MS (2016). Study of myringoplasty in wet and dry ears in mucosal type of chronic otitis media. J Clin Diagn Res..

[20] O’Reilly BJ, Cheverton EB, Wylie I, Thakkar C, Butler P, Sathanathan N (1991). The value of CT scanning in chronic suppurative otitis media. J Laryngol Oto..

[21] Thukral CL, Singh A, Singh S, Sood AS, Singh K (2015). Role of high resolution computed tomography in evaluation of pathologies of temporal bone. J Clin Diagn Res..

[22] Karki S, Pokharel M, Suwal S, Poudel R (2017). Correlation between preoperative high resolution CT findings with surgical findings in chronic otitis media squamosal type. Kathmandu Univ Med J..

[23] Feeney MP, Grant IL, Mills DM (2009). Wideband energy reflectance measurements of ossicular chain discontinuity and repair in human temporal bone. Ear Hear..

[24] Voss SE, Horton NJ, Woodbury RR, Sheffield KN (2008). Sources of variability in reflectance measurements on normal cadaver ears. Ear Hear..

[25] Voss SE, Merchant GR, Horton NJ (2012). Effects of middle-ear disorders on power reflectance measured in cadaveric ear canals. Ear Hear..

[26] Voss SE, Rosowski JJ, Merchant SN, Peake WT (2001). Middle-ear function with tympanic-membrane perforations Measurements and mechanisms. J Acoust Soc Am..

[27] Voss SE, Rosowski JJ, Merchant SN, Peake WT (2001). How do tympanic-membrane perforations affect human middle-ear sound transmission?. Acta Otolaryngol..

[28] Shahnaz N, Bork K (2006). Wideband reflectance norms for Caucasian and Chinese young adults. Ear Hear.

